# Suppression of Bacterial Leaf Spot by Green Synthesized Silica Nanoparticles and Antagonistic Yeast Improves Growth, Productivity and Quality of Sweet Pepper

**DOI:** 10.3390/plants10081689

**Published:** 2021-08-17

**Authors:** Eman F. A. Awad-Allah, Amany H. M. Shams, Amira A. Helaly

**Affiliations:** 1 Soil and Water Sciences Department, Faculty of Agriculture, Alexandria University, Alexandria 21545, Egypt; 2 Plant Pathology Department, Faculty of Agriculture, Alexandria University, Alexandria 21545, Egypt; amany.shams@alexu.edu.eg; 3 Vegetable Crops Department, Faculty of Agriculture, Alexandria University, Alexandria 21545, Egypt; amira.helaly@alexu.edu.eg

**Keywords:** bacterial spot disease, bio-control yeast, Nano-SiO_2_, nanotechnology, sweet pepper, sustainability

## Abstract

Plants are challenged with many kinds of biotic stresses caused by different living organisms, which result in various types of diseases, infections, and damage to crop plants and ultimately affect crop productivity. Plant disease management strategies based on current approaches are necessary for sustainable agriculture. A pot experiment was carried out under greenhouse conditions to evaluate the potential of green synthesized silica nanoparticles (SiO_2_-NPs) and antagonistic yeast (*Saccharomyces cerevisiae*) against pepper bacterial leaf spot disease, caused by *Xanthomonas vesicatoria*. In addition, to assess their efficacy and suppressive effects in reducing disease severity and improving sweet pepper growth, productivity, and quality. Results revealed that the combination of BCA (5%) and SiO_2_-NPs (150 ppm) was the most effective treatment for reducing disease severity and improving vegetative growth characters, mineral contents (N, P, K, Ca, Mg, and Si in leaves), as well as stimulating polyphenol oxidase (PPO) activity of sweet pepper leaves at 90 days from transplanting, while also at harvesting time enhancing sweet pepper fruit yield quality parameters significantly. In conclusion, green synthesized silica nanoparticles combined with antagonistic yeast have the potential to suppress a bacterial leaf spot disease with ecologically-sound management, while also boosting sweet pepper growth, productivity, and quality.

## 1. Introduction

Bacterial leaf spot (BLS) of sweet pepper (*Capsicum annuum* L.), caused by *Xanthomonas vesicatoria*, is the most common and serious disease worldwide [1,2,3,4]. The gram-negative bacteria *X. vesicatoria* can infect all above-ground plant parts, causing early defoliation and necrotic lesions on leaves, stems, and fruits [1,2]. In Egypt, bacterial spot disease is a devastating and economically significant disease affecting tomato and pepper plants [5]. Sweet pepper productivity and fruit quality were both reduced by this disease, resulting in significant economic losses [2,4]. Bacterial spots are most common in warm, humid climates, as well as in greenhouses [6]. Pathogens enter plants through natural openings (such as stomata) as well as wounds. Warm, wet weather promotes disease development [6]. Wind-driven rain can exacerbate disease severity by splashing pathogens onto healthy leaves and fruits [6]. Contamination of seeds may also be an important source of inoculum [5]. As a result, pepper growers must be proactive in combating bacterial spot disease by employing efficient, sustainable management strategies [3].

Nanotechnology has a great potential in the agricultural sector because it can provide eco-friendly alternatives to various agrochemicals [7,8]. The efficacy of silica nanoparticles (SiO_2_-NPs) applications in agriculture is increasing rapidly, which can help mitigate both biotic and abiotic stresses, providing new solutions to problems in plants and crop science to enhance the quality of plant products more efficiently [9]. Many agricultural wastes, such as rice husk, barley grass waste, and sugarcane bagasse, can be processed and used as a raw material for green synthesis of SiO_2_-NPs, conserving environment quality and lowering the agriculture sector’s carbon footprint [10,11]. The small size, greater surface area, higher solubility, and surface reactivity of SiO_2_-NPs are unique physico-chemical features that result in greater and easier nutrient absorption by plants [12]. The potential of SiO_2_-NPs applications in plant disease management and the associated mechanisms have not been explored thoroughly and further research needs to be carried out to investigate the effectiveness of SiO_2_-NPs in promoting plant growth under abiotic stresses.

Biological control refers to the use of biological agents to a host plant in order to control disease development by a pathogen [13]. Biological control of plant diseases has been considered as a viable alternative to chemical control [14]. These biological control activities are carried out either directly through antagonism of pathogens or indirectly by induction a plant-mediated resistance response [13,14]. Yeasts occur in all environments and have been reported as effective antagonists of various plant pathogens [15]. Many of these unicellular fungi have been explored for biocontrol applications due to their antagonistic ability, low cultivation requirements, and low biosafety concerns [15]. Five yeast species (*Candida oleophila*, *Aureobasidium pullulans*, *Metschnikowia fructicola*, *Cryptococcus albidus*, and *Saccharomyces cerevisiae*) are currently or have been registered for application as plant protection agents or biocontrol products [15]. Understanding the mechanisms of biological control of plant diseases through antagonist-pathogen interactions may help to select more effective bio-control agents (BCA) for sustainable plant disease management [14,15].

Macro- and micro-nutrients are commonly used to increase crop yields and improve overall plant health and quality, and their judicious application in agriculture is vital for increased production efficiency and a sustainable ecosystem [16,17]. Silicon (Si) is a mineral nutrient that has a significant impact on plant diseases, and in many situations, it is the first line of defense against disease [18]. Therefore, the main objective of this study was to evaluate the effect of green synthesized silica nanoparticles (SiO_2_-NPs) and antagonistic yeast (*Saccharomyces cerevisiae*) on pepper bacterial leaf spot disease, caused by *Xanthomonas vesicatoria*. As well, to evaluate their efficacy and suppression effects to reduce disease severity and improve growth, productivity and quality of sweet pepper.

## 2. Materials and Methods

A pot experiment was conducted under greenhouse conditions at Plant Pathology Department, Faculty of Agriculture, Alexandria University, Egypt, to evaluate the potential of green synthesized silica nanoparticles (SiO_2_-NPs) and antagonistic yeast (*Saccharomyces cerevisiae*) against pepper bacterial leaf spot disease, caused by *Xanthomonas vesicatoria*. As well, to evaluate their efficacy and suppression effects to reduce bacterial spot disease severity and improve growth, productivity and quality of sweet pepper.

### 2.1. Samples Collection and Isolation of the Associated Bacteria

**Figure 1:**
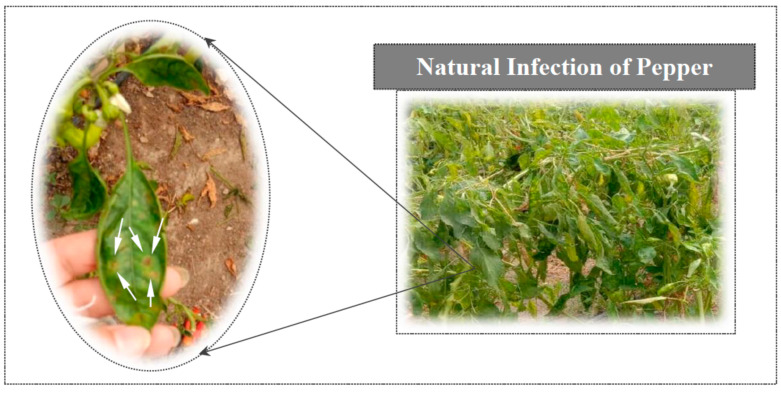
Pepper plant leaves with bacterial spot symptoms have brown lesions surrounded by yellow halos, from which bacteria were isolated.

Naturally infected pepper leaves showing typical symptoms of bacterial spot disease were collected from the Experimental Station of the Faculty of Agriculture, Alexandria University, in Alexandria governorate, Egypt during 2019 season as shown in Figure 1.

**Figure 2:**
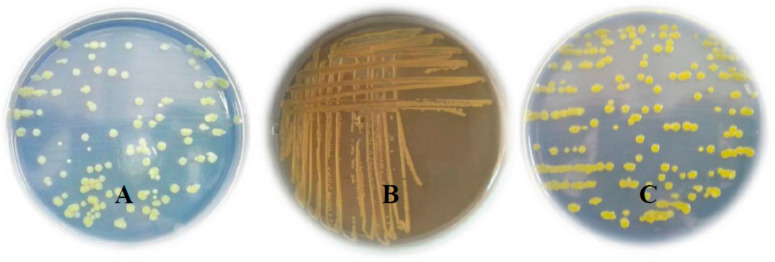
Colonial morphology on: (**A**) Nutrient agar medium, (**B**) Peptone Sucrose Agar (PSA) medium, and (**C**) Glycerol agar medium.

The infected pepper leaves were first washed with tap water to remove soil dust and then surface-sterilized with 1% sodium hypochlorite solution (NaOCl) for 3 min, followed by two successive rinses in sterile water. Each bacterial lesion was put into a sterilized mortar and homogenized in 0.2 mL of sterilized water, then left to stand for 20 min. The resulting suspension was then streaked onto plates containing nutrient agar media. The associated bacterial colonies were purified and streaked onto different simi-selective media as shown in Figure 2. Characterization of the bacteria (3 isolates) from symptomatic tissues were performed based on classical and molecular methods [6,19]. After pathogenicity test of the 3 isolates (data not shown), one aggressive isolate was selected for sequence analysis of 16S rRNA [20].

### 2.2. Molecular Identification of Isolated Bacteria

DNA extraction was carried out according to Ausubel et al., [21]. Bacterial isolates were grown overnight in Luria-Bertani (LB) medium at 28 °C with constant shaking at 200 rpm. Cells from 3 mL culture were pelleted by centrifugation at 6000× *g* for 5 min by using a Hermle Z230M microcentrifuge. Cells of each culture were washed in TE buffer (10 mM Tris-HCl, 1 mM EDTA, pH 8.0), then resuspended in a mixture of 567 μL Tris EDTA, 30 μL of 10% Sodium Dodecyl Sulphate (SDS) and 3 μL proteinase K (20 mg mL^−1^). After incubation at 37 °C for 1 h, 100 µL 5 M NaCl and 80 µL of CTAB/NaCl solution were added and the tubes were inverted well before incubation for 10 min in a water bath at 65 °C. Phenol/chloroform/isoamyl alcoholic mixture (0.8 mL) was then added, mixed thoroughly and the tubes were centrifuged at 11,000× *g* for 5 min. The aqueous supernatant was then taken, and the phenol/chloroform/isoamyl step was repeated one more time. DNA was precipitated by adding equal volume of isopropanol, and washed with 70% ethanol and air dried. DNA pellets were suspended in 100 µL sterilized distilled water [22].

PCR amplification of 16S rRNA gene was carried out. Full length (1550 bp) of 16S rRNA gene was amplified from 13 isolates using two primers: P0 (5′-GAAGAGTTTGATCCTGGCTCAG-3′) and P6 (5′-CTACGGCTACCTTGTTACGA-3′). PCR amplification was carried out in a total volume of 25 µL containing 12.5 µL Dream Taq Green PCR master mix kit, 0.5 µL of 10 pmol forward primer (P0), 0.5 µL of 10 pmol reverse primer (P6), 3 µL 50 ng of bacterial genomic DNA [23]. PCR amplification was performed in a thermal cycler (Techne, UK) programmed for one cycle at 95 °C for 5 min followed by 34 cycles each with 45 s at 95 °C for denaturation, 1 min at 50 °C for annealing and 2 min at 72 °C for elongation. Reaction mixture was then incubated at 72 °C for 10 min for final extension. PCR products were electrophoretically separated on a 1.5% agarose gel in TBE buffer according to Maniatis et al., [24], stained ethidium bromide solution and photographed under UV light.

For sequencing of 16S rRNA gene and alignment, the amplified product (1550 bp) of 16S rRNA was sequenced by Big Dye terminator cycle sequencing kit. Sequencing products were purified using Centri-Sep spin columns and were resolved on the ABI PRISM model^®^ 310 automated DNA sequencer at the Lab Technology Scientific Services Company. A search in the GenBank database to identify the bacteria was achieved in a BLAST search at the National Center for Biotechnology Information (NCBI) web site (http://www.ncbi.nlm.nih.gov (accessed on 12 July 2021)). The search revealed that the sequence corresponding to bacterial leaf spot identical (96% homology) to that of *Xanthomonas vesicatoria*. The Genbank accession numbers of the bacterial isolate was MZ501569.

### 2.3. Green Synthesis of Silica Nanoparticles (SiO_2_-NPs)

**Figure 3:**
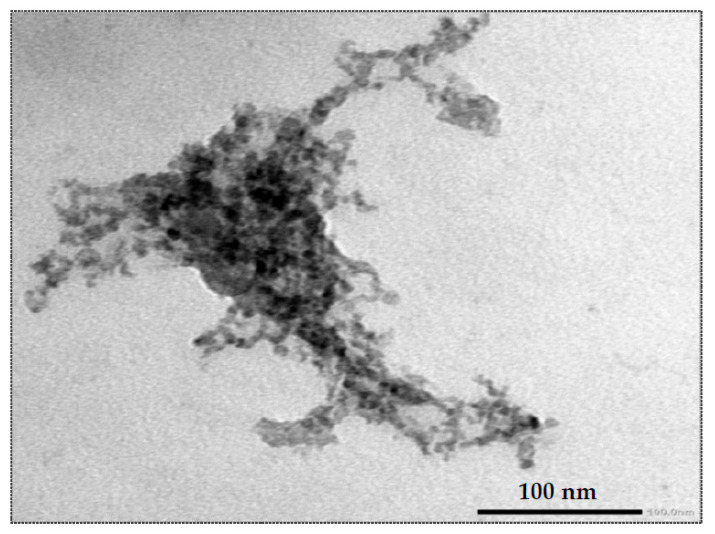
Transmission electron microscopy (TEM) image of silica nanoparticles (SiO_2_-NPs).

Synthesis of silica nanoparticles (SiO_2_-NPs) was achieved with slight modifications of Yuvakkumar et al., [25] protocol. The useless materials, rice husks (RHs), were washed thoroughly with distilled water to remove any dust or other adhering impurities. The washed RHs were air-dried at room temperature and then dried in the oven at 100 °C for 24 h. The obtained rice husk ash (RHA) was then crushed to powder form by using a miller. The RHA powder was refluxed with 6N HCl for 2 h, and then filtered and washed with deionized water in order to extract pure nano-silica. The produced SiO_2_-NPs were obtained by calcining HCl-treated RHs in a muffle furnace at 700 °C for 5 h. The sample was analyzed by transmission electron microscope (TEM) examination by placing the synthesized SiO_2_-NPs on a carbon-coated copper grid and left for drying at room temperature before being characterized via TEM instrument (JEM-1400 Plus; JEOL, Tokyo, Japan). For evaluation of the particle size and distribution of SiO_2_-NPs, the selected area electron diffraction (SAED) was utilized to investigate the nature of the prepared nanoparticles (SiO_2_-NPs) in terms of their amorphous state as shown in Figure 3. The SiO_2_-NPs suspensions with a diameter of ~50–70 nm were used for the dosing plants.

### 2.4. Experimental Design and Treatments

In this study, the seeds of sweet pepper (*Capsicum annuum* (L.) *cv.* Hybrid 702) were sown in the nursery using foam trays on 15 August 2020, and cared by regular practices for seedlings production in greenhouse. After 4 weeks, uniform pepper seedlings (4–5 leaves) were transplanted into pots (30 cm inner diameter) containing 8 kg mixture of sterilized clay and sand (2:1 *v*/*v*), with two plants/pot under greenhouse conditions at 23/20 °C ± 2 day/night temperature, and 76–80 % relative humidity at Plant Pathology

Department, Faculty of Agriculture, Alexandria University, Egypt, during the growing season of 15th September 2020. According to the recommended doses of agricultural practices, nitrogen (N) as ammonium sulfate (20.5% N) at 2.5 g/pot, phosphorus (P) as calcium superphosphate (15.5% P_2_O_5_) at 1.5 g/pot and potassium (K) as potassium sulfate (48% K_2_O) at 1 g/pot were added to each pot before transplanting. Also, further N doses (ammonium sulfate 20.5% N) were added at 30, 60, and 90 days after transplanting at 1.5 g/pot. The pots were irrigated on three days frequency.

The experiment treatments were arranged in a Randomized Complete Block Design (RCBD) with five replicates per treatment. Twenty-four treatments were divided into, 12 treatments without bacterial infection and 12 treatments were infected with bacteria (*X. vesicatoria*), under greenhouse conditions. Sweet pepper plants were sprayed with aqueous solutions of SiO_2_-NPs (0, 50, 100, and 150 ppm), antagonistic yeast extract (0, 3, and 5%), or in combinations of both SiO_2_-NPs and yeast extract. Control plants were sprayed with sterilized water. Exogenous applications of SiO_2_-NPs (0, 50, 100, and 150 ppm) were applied using hand atomizer. Five drops of 80% Tween^®^ 20 were used with every prepared solution to maximize dissemination on pepper leaves. All foliar treatments of SiO_2_-NPs were applied four times at 30, 45, 60, 75 days from transplanting, as well as control plants (sprayed with sterilized tap water). Antagonistic yeast extract (*Saccharomyces cerevisiae*) was used as biocontrol agent (BCA) against pepper bacterial leaf spot disease, caused by *X. vesicatoria*. Three levels of antagonistic yeast extract namely 0, 3, and 5% considered as BCA_0_, BCA_3_, and BCA_5_, respectively, were applied five times during the growing season of sweet pepper plants, and the first application was 20 days from transplanting and repeated each 21 days intervals. The plants were sprayed using an ordinary sprayer with a sharp nozzle, with uniform coverage until run-off, with a wetting agent Tween^®^ 20 (0.1%) added to the spraying solution. Control plants were sprayed with fresh sterilized water. Yeast extract treatments were prepared from active dry yeast (*Saccharomyces cerevisiae*) according to the modified method of Francesca et al., [26] by dissolving amount of dry yeast in water followed by adding sugar (as a source of C and N) at a ratio of 1:1 and kept 24 h in a warm place for activation before application on the plants. Moreover, yeast extract is a rich source in beneficial bioconstituents such as amino acids, peptides, phytohormones, vitamins, carbohydrates, trace elements, and other growth factors….etc, hence making it suitable for foliar application. The nutritional contents of the yeast extract according to Awad-Allah et al., [27], are shown in the Table 1.

For inoculation purposes, *X. vesicatoria* strain was grown overnight in LB broth, collected by centrifugation (15,000× *g* for 15 min), and resuspended in sterile water. The bacterial concentration was adjusted to 10^8^ colony-forming units per mL (CFU/mL) and plants were spray inoculated 3 days after the first treatment (i.e., SiO_2_-NPs and antagonistic yeast extract) applications. Inoculated plants were kept on a greenhouse bench and recorded for foliar bacterial spot disease severity assessment 4 weeks after inoculation.

### 2.5. Measurements

#### 2.5.1. Disease Incidence and Severity

Disease severity was recorded using the following scale according to Le et al., [28]: 1 = symptomless, 2 = a few necrotic spots on a few leaflets, 3 = a few necrotic spots on many leaflets, 4 = many spots with coalescence on few leaflets, 5 = many spots with coalescence on many leaflets, 6 = severe disease and leaf defoliation, and 7 = plant dead. The disease severity scale was used based on leaf spot disease development in which infected plants were recorded in each replicate.

#### 2.5.2. Vegetative Growth Parameters

Five pepper plants were chosen randomly from each treatment at 90 days after transplanting, the vegetative growth characters of sweet pepper plant were recorded: plant height (cm), number of leaves per plant, and number of branches per plant.

#### 2.5.3. Polyphenol oxidase Activity Measurements

Polyphenol oxidase (PPO) activity was determined by using a spectrophotometric method according to the procedure method given by Mayer et al. [29]. One gram of plant leaves was homogenized in 2 mL of 0.1 M sodium phosphate buffer (pH 6.5) at 4 °C. The homogenate was centrifuged at 10,000× *g* for 15 min. The supernatant served as enzyme source and polyphenol oxidase activity was determined. The reaction mixture consisted of 1.5 mL of 0.1 M sodium phosphate buffer (pH 6.5) and 200 μL of the enzyme extract. To start the reaction, 200 μL of 0.01 M catechol was added and the activity was expressed as change in absorbance at 495 nm at 30-s intervals for 3 min. The enzyme activity was expressed as change in absorbance (ΔOD) min^−1^ g^−1^ of fresh tissue.

#### 2.5.4. Leaf Chemical Composition

Plant leaves were oven dried at 70 °C for 48 h, and N, P, K, Ca, Mg and Si contents were estimated. Total nitrogen content was determined according to the method described by Jones Jr, [30]. Total phosphorus content was measured according to Page, et al., [31]. While, total potassium content was determined according to the method described by Jones Jr, [30]. Also, calcium and magnesium contents were measured according to Jackson [32]. For silicon (Si) analysis, a spectrometric method was used for determining Si in leaves tissue according to adapted method described by Liang et al., [33].

#### 2.5.5. Fruit Yield and Quality

At harvesting time, samples of sweet pepper fruits were randomly harvested from each treatment to measure fruit length (cm), fruit diameter (cm), fruit number per plant, and fruit weight per plant.

### 2.6. Statistical Analysis

The data obtained were subjected to analysis of variance (ANOVA) according to Gomez and Gomez [34], using CoStat computer software [35], (CoHort Software version 6.303, Monterey, CA, USA), and LSD at 0.05 level of significance was used for the comparison between means.

## 3. Results

The major goal of this work was to evaluate the potential of green synthesized silica nanoparticles (SiO_2_-NPs) and antagonistic yeast (*Saccharomyces cerevisiae*) against *Xanthomonas vesicatoria*-caused pepper bacterial leaf spot disease. In addition, to assess their efficacy and suppressive effects in reducing disease severity and improving sweet pepper growth, productivity, and quality.

**Figure 4:**
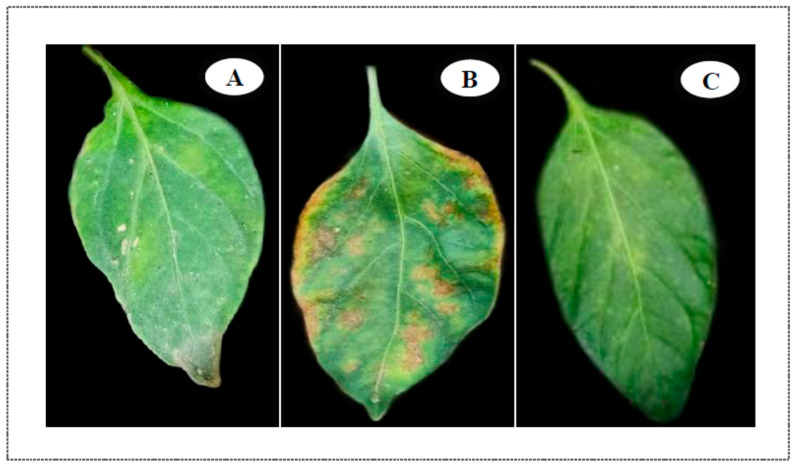
Sweet pepper plant leaves with inoculation and treatments of SiO_2_-NPs and BCA. (**A**) Control, (**B**) Artificial inoculation symptoms caused by *Xanthomonas vesicatoria*, (**C**) Suppression of pepper bacterial leaf spot disease after treatments with (150 ppm SiO_2_-NPs and 5% BCA).

Table 2 shows the incidence and severity of bacterial spot disease on sweet pepper leaves after infection, as affected by different treatments of SiO_2_-NPs (ppm), and BCA (%). Exogenous foliar sprays of SiO_2_-NPs (ppm) and BCA (%) on greenhouse-grown sweet pepper plants consistently reduced bacterial spot severity as compared with untreated, infected control plants. Also, our results revealed that the interaction between BCA at 5% and SiO_2_-NPs at 150 ppm, was the best interaction treatment for effectively reducing disease severity. Figure 4 shows sweet pepper plant leaves with inoculation and foliar treatments of SiO_2_-NPs and BCA. The suppressive effects of SiO_2_-NPs (150 ppm) and BCA (5%) treatments on pepper bacterial leaf spot disease are shown in Figure 4C. In addition, the treated plants appeared to be in good health and exhibited no signs of BLS disease. As a result, green synthesized silica nanoparticles (SiO_2_-NPs) and antagonistic yeast (*Saccharomyces cerevisiae*) can enhance sweet pepper resistance against bacterial leaf spot disease, caused by *X. vesicatoria.*

**Figure 5:**
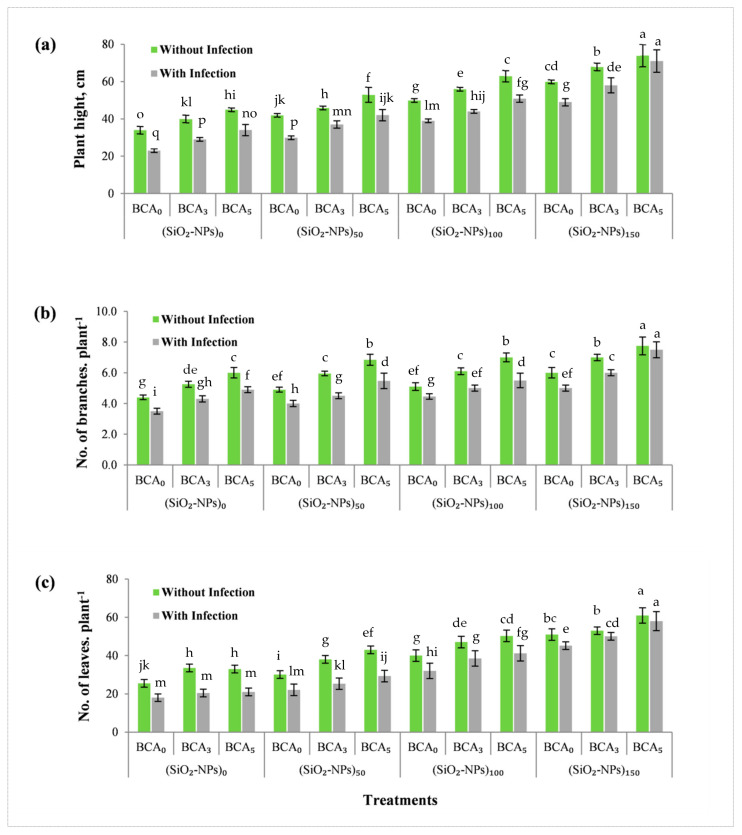
Vegetative growth parameters of sweet pepper at 90 days after transplanting, as affected by different treatments of SiO_2_-NPs (ppm), and BCA (%) without and with bacterial infection. (**a**) plant height, (**b**) number of branches per plant, and (**c**) number of leaves per plant. Error bars represent the mean ± standard deviation (SD) of the data of 5 replications. Different letter(s) above the error bars indicate statistically significant differences at (*p* ≤ 0.05).

Figure 5 shows the effect of different treatments of SiO_2_-NPs (ppm), BCA (%), and their interactions without and/or with bacterial infection on the studied vegetative growth parameters, such as plant height, number of branches per plant, and number of leaves per plant at 90 days after transplanting. The obtained results showed that vegetative growth parameters were significantly increased with increasing BCA and SiO_2_-NPs levels without and/or with bacterial infection. In sweet pepper plants with bacterial leaf spot infection, treatments with BCA and SiO_2_-NPs were found to induce significant recovery for the reduction in vegetative growth parameters. The interaction effect between foliar sprays of SiO_2_-NPs (ppm) and BCA (%) had significant effect on different vegetative growth parameters than control treatment without and/or with bacterial infection. In this respect, sweet pepper plants which sprayed with SiO_2_-NPs (150 ppm) and BCA (5%) treatments resulted in the highest values of plant height as well as number of branches per plant, and number of leaves per plant at 90 days after transplanting.

**Figure 6:**
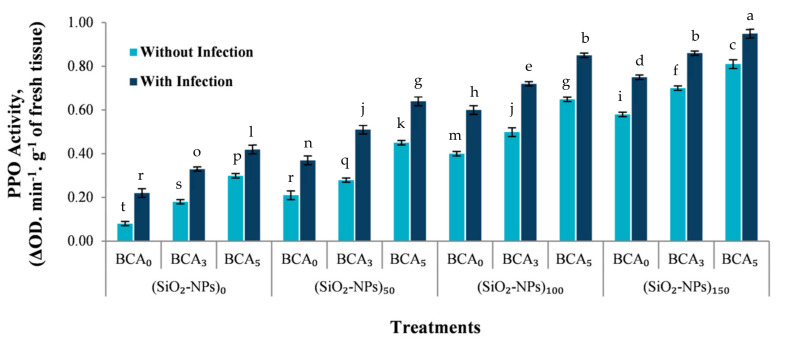
Polyphenol oxidase (PPO) activity, (change in absorbance min^−1^ g^−1^ of fresh tissue), of sweet pepper leaves as influenced by different treatments of SiO_2_-NPs (ppm), and BCA (%) without and with bacterial infection. Error bars represent the mean ± SD of the data of 5 replications. Different letter(s) above the error bars indicate statistically significant differences at (*p* ≤ 0.05).

The effect of different treatments of SiO_2_-NPs (ppm), and BCA (%) without and with bacterial infection was also investigated on polyphenol oxidase (PPO) activity, (change in absorbance min^−1^ g^−1^ of fresh tissue), of sweet pepper leaves at 90 days from transplanting (Figure 6). The results suggested that BCA and SiO_2_-NPs have the potential to stimulate the activity of polyphenol oxidase (PPO) in sweet pepper leaves while also promoting the growth of sweet pepper plant. The combination of BCA (5%) and SiO_2_-NPs (150 ppm) was the most effective treatment for stimulating polyphenol oxidase (PPO) activity and therefore reducing disease severity of sweet pepper leaves at 90 days from transplanting. It seems that polyphenol oxidase enzyme can boost plant resistance against pathogens and may play an important role in sweet pepper defense mechanisms against bacterial leaf spot disease caused by *Xanthomonas vesicatoria*.

Data in Table 3 show leaf chemical composition, (%), of sweet pepper plants as affected by different treatments of SiO_2_-NPs (ppm), and BCA (%) without and with bacterial leaf spot (BLS) infection. It is obvious that leaf chemical composition of sweet pepper plants, such as N, P, K, Mg, Ca, and Si contents, was significantly affected by sprayed plants with different treatments of SiO_2_-NPs (ppm) and BCA (%) without and with bacterial leaf spot (BLS) infection than the control treatment. In this respect, sweet pepper plants which sprayed with SiO_2_-NPs (150 ppm) and BCA (5%) treatments achieved the highest concentrations of N, P, K, Mg, Ca and Si in leaves of sweet pepper plants at 90 days after transplanting.

**Figure 7:**
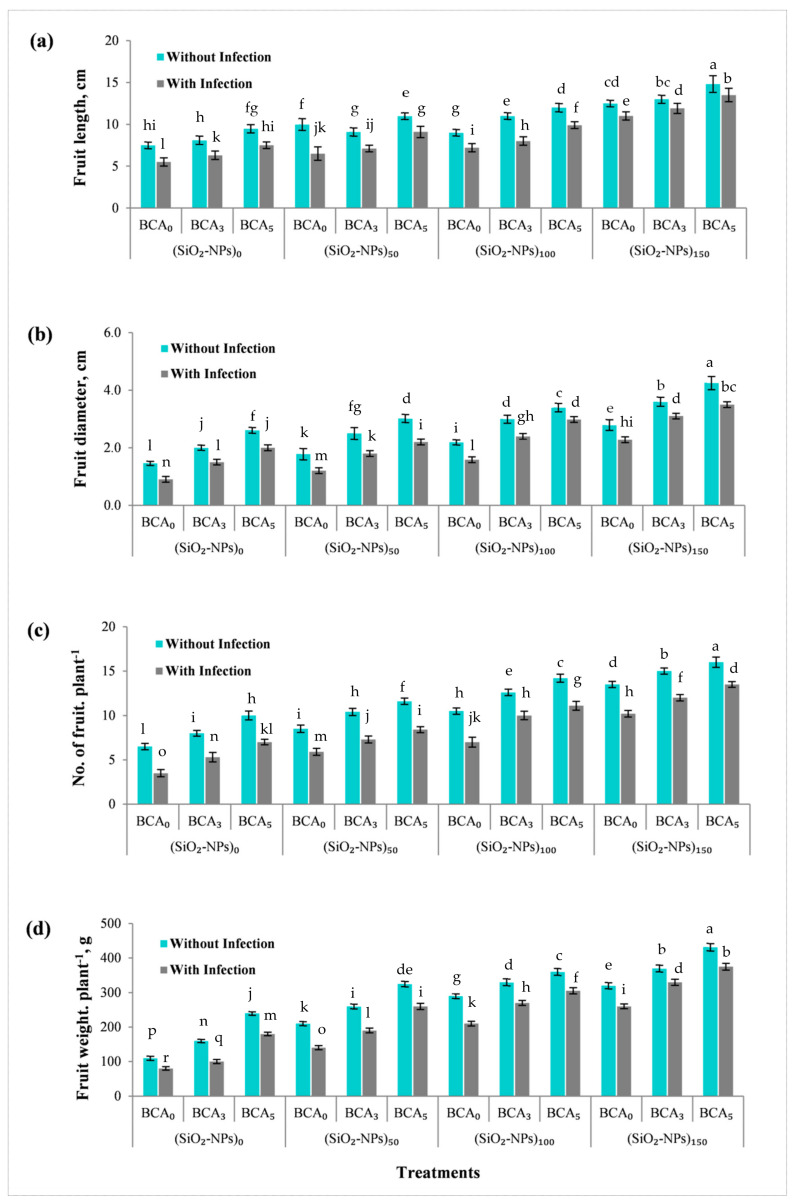
Pepper fruit yield and quality parameters at harvesting time, as affected by different treatments of SiO_2_-NPs (ppm), and BCA (%) without and with bacterial infection. (**a**) fruit length, (**b**) fruit diameter, (**c**) fruit number per plant, and (**d**) fruit weight per plant. Error bars represent the mean ± SD of the data of 5 replications. Different letter(s) above the error bars indicate statistically significant differences at (*p* ≤ 0.05).

Figure 7 shows the effect of different treatments of SiO_2_-NPs (ppm), BCA (%), and their interactions without and/or with bacterial infection on sweet pepper fruits quality parameters, such as fruit length (cm), fruit diameter (cm), fruit number per plant, and fruit weight per plant at harvesting time. The obtained results from Figure 7 showed that increasing SiO_2_-NPs (ppm) and BCA (%) treatments without and/or with bacterial infection at harvesting time significantly increased all sweet pepper quality parameters.

## 4. Discussion

Plant disease management strategies based on current approaches are necessary for sustainable agricultural production. In this study, we investigated the effectiveness of foliar sprays of green synthesized silica nanoparticles (SiO_2_-NPs) and antagonistic yeast (*Saccharomyces cerevisiae*) against pepper bacterial leaf spot disease, caused by *Xanthomonas vesicatoria*. In addition, their efficacy and suppressive effects in reducing disease severity and boosting sweet pepper growth, productivity, and quality will also be evaluated.

In the greenhouse, pepper plants treated with SiO_2_-NPs, BCA, or a combination of SiO_2_-NPs and BCA consistently had less bacterial spot as compared with untreated, infected control plants (Table 2). Furthermore, the best interaction treatment for effectively suppressing disease severity was the combination of BCA at 5% and SiO_2_-NPs at 150 ppm (Figure 4). Also, treated plants looked healthy and showed no symptoms of BLS disease. As noted in the introduction, pepper growers must be proactive in their approach to bacterial spot disease resistance by implementing effective, long-term management options. Furthermore, SiO_2_-NPs and BCA have the potential to be a low-cost, high-efficiency, safe, and sustainable alternative for plant disease protection.

Silicon is an essential element for some plants and quasi-essential for many others and regulates a range of physiological processes including germination, vegetative growth, photosynthesis, and stress tolerance [36]. As a result, assessing the effects of silica nanoparticles (SiO_2_-NPs) on various physiological processes is critical, as SiO_2_-NPs are considered to be more efficient than bulk particles due to their tiny size, high surface area, and reactivity [37]. SiO_2_-NPs can promote plant growth and plant resistance against biotic [38], and abiotic [39] stresses. In this study according to TEM examination, the SiO_2_-NPs suspensions used for plant dosing were well dispersed, with the primary particle size of 60 ± 8 nm (average ± standard deviation). SiO_2_-NPs have a spherical shape and a particle size that is nearly consistent. The lack of a stabilizing agent and the high specific surface area promote the tendency of particles to agglomerate in cluster form, resulting in minimal agglomeration of these very small particles. However, these cluster agglomerated particles remain small, not exceeding 70 nm under all agglomeration conditions. The interactions between SiO_2_-NPs and plant leaves were studied [38], confirming that SiO_2_-NPs with a size range of ~50–70 nm were able to enter the leaf through the stomata and spread across the large extracellular air spaces of the spongy mesophyll without penetrating any cell walls. The spongy mesophyll, an attractive target, is used for prolonged and sustained release of SiO_2_-NPs, and therefore plants developed resistance against bacterial pathogen. Furthermore, the results demonstrated that SiO_2_-NPs stimulated the release of salicylic acid, a defense-related plant hormone that activated the immune response of plants to protect them from pathogen attacks. It’s also worth noting that systemic acquired resistance (SAR) was successfully created in a concentration-dependent manner between 20 and 320 mg L^−1^ SiO_2_-NPs, however, with a higher dose led to a detrimental effect on SAR induction [38,40].

Our results show that vegetative growth parameters were significantly increased with increasing BCA and SiO_2_-NPs levels without and/or with a bacterial infection versus control plants (Figure 5). In many studies, the simulative effect of yeast, which is environmentally friendly, nutritious, and convenient to use, has been found to accelerate cell division, elongation, enlargement, chlorophyll production, protein, and nucleic acid synthesis of plants [41,42,43]. The nutritional content of yeast extract, which includes a relatively higher proportion of amino acids, a higher percentage of peptides, phytohormones, higher values of vitamins, carbohydrates, trace elements, and other growth factors, may explain why plants respond better to foliar application of yeast extract (Table 1 and Figure 5). Furthermore, a preliminary experiment study of *S. cerevisiae* antagonistic activity against *X. vesicatoria* in Petri dishes revealed that *S. cerevisiae* had an antagonistic effect against *X. vesicatoria* (data not shown). Also, the obtained results showed that foliar spray of antagonistic yeast can stimulate the activity of polyphenol oxidase (PPO) in sweet pepper leaves while also promoting the growth and plant resistance against bacterial pathogen. As a result, *S. cerevisiae* can therefore either directly reduce BLS disease by antagonizing the bacterial pathogen or indirectly by inducing a plant-mediated resistance response in sweet pepper leaves by stimulating the activity of polyphenol oxidase (PPO).

Plants react to bacterial pathogens by activating a range of defense responses linked to the accumulation of several factors, including antioxidant defense enzymes and pathogens inhibitors [44]. These results in changes to the metabolism of cells, particularly in enzymes such as polyphenol oxidase (PPO), peroxidase (POX), catalase (CAT), superoxide dismutase (SOD), and... etc, due to the interaction of the pathogen and the host plant [45]. Also, all of these enzymes have been shown to be involved in the development of plant resistance to bacterial spot and can be used as biochemical markers of host resistance [46]. Similarly, PPO expression could be used as a biochemical marker to predict the outcome of the interaction between different genotypes and the pathogens that cause bacterial leaf spot disease [47].PPO, the nuclear encoded enzyme, catalyzes oxygen-dependent phenols oxidation to quinones and, if the plant is injured or infected, PPO levels increase in the plant [48]. No clear report has been presented on how PPO might have an effect on pathogens, but several mechanisms have been identified to affect PPO on the pathogens, including the direct antibiotic and cytotoxic activities to pathogens of quinones generated by PPO, cross-linkages between PPO-generated quinones with phenolic compounds, and proteins which could lead to the development of physical barrier against pathogens [45]. The current study therefore showed that increasing polyphenol oxidase activity can boost the resistance of sweet pepper plants to *Xanthomonas vesicatoria*-induced bacterial leaf spot disease. Several studies have shown that after pathogen infection, POX, CAT, PPO, and SOD enzyme activity increased in resistant cultivars compared to susceptible cultivars, implying that antioxidant enzymes and isoforms can mitigate bacterial spot disease-induced biotic stress in *Capsicum annuum* L. cultivars [49,50].

According to the findings of this study, the interaction between BCA at 5% and SiO_2_-NPs at 150 ppm can be used to manage the bacterial leaf spot disease of sweet pepper. As a result, this study encourages the use of these treatments in the management of other plant diseases. The current study is an experiment in the new field of biological management of plant diseases by antagonistic yeast. This study also confirmed that effective bio-control agents (BCA) and SiO_2_-NPs can directly antagonize plant pathogens and indirectly inhibit plant pathogens by increasing systemic induced resistance by stimulating the activity of polyphenol oxidase (PPO) enzyme.

Silica nanoparticles have emerged as a promising tool for boosting plant growth and productivity, as well as disease management. At the same time, nano-SiO_2_ deposition in leaf tissue improves plant defense against pathogens [51]. In addition, silicon-mediated acquisition, uptake, and translocation of nutrients such as nitrogen (N), phosphorus (P), potassium (K), calcium (Ca), magnesium (Mg), sulfur (S), iron (Fe), zinc (Zn), manganese (Mn), copper (Cu), and boron (B) under both deficiency and excess conditions [52]. Silicon can also increase plant resistance to bacterial pathogens by increasing the activity of the Polyphenol oxidase (PPO) enzyme in plant leaves [53].

In summary, our results revealed that green synthesized silica nanoparticles (SiO_2_-NPs) and antagonistic yeast (*Saccharomyces cerevisiae*) can enhance sweet pepper resistance against bacterial leaf spot disease, caused by *Xanthomonas vesicatoria*. Furthermore, environmentally sound bacterial pathogen management was implemented, as well as improved sweet pepper growth, productivity, and quality. As a result, the combination treatment of foliar spray with SiO_2_-NPs (150 ppm) and BCA (5%) could be recommended for increasing sweet pepper resistance against bacterial leaf spot disease and improving pepper growth, productivity, and quality cultivated under similar conditions to this study. However, further experiments are needed to explore higher levels of SiO_2_-NPs (ppm) and BCA (%), and their effects on plant diseases. Also, the potential of these treatments for controlling bacterial leaf spot should be further investigated and explored under field conditions prior to its practical usage.

## 5. Conclusions

The most common and serious disease of sweet pepper (*Capsicum annuum* L.) is bacterial leaf spot (BLS), which is caused by *Xanthomonas vesicatoria*. As a result, pepper growers must be proactive in their approach to bacterial spot disease resistance by employing efficient, sustainable management strategies.

In this study, green synthesized silica nanoparticles (SiO_2_-NPs) and antagonistic yeast (*Saccharomyces cerevisiae*) were shown to boost sweet pepper resistance against bacterial leaf spot disease. Our results also revealed that the combination of BCA (5%) and SiO_2_-NPs (150 ppm) was the most effective treatment for reducing disease severity and improving vegetative growth characters such as plant height, number of leaves per plant, number of branches per plant, as well as enhancing mineral contents (N, P, K, Ca, Mg and Si in leaves) and stimulating polyphenol oxidase (PPO) activity of sweet pepper leaves at 90 days from transplanting, while also at harvesting time enhancing sweet pepper fruit yield quality parameters such as fruit length, fruit diameter, fruit number per plant, and fruit weight per plant.

In conclusion, green synthesized silica nanoparticles and antagonistic yeast have the potential to reduce the susceptibility of sweet pepper plants to bacterial leaf spot disease with ecologically-sound management, while also improving sweet pepper growth, productivity, and quality.

## Figures and Tables

**Table 1:** The nutritional contents of the yeast extract *.

Minerals (mg/g)		Amino Acids (mg/100 g)		Vitamins (mg/100 g)	
K	23.0 ± 0.05 ^¶^	Arginine	2.18 ± 0.07	Vitamin B1	3.25 ± 0.03
P	16.0 ± 0.12	Aspartic acid	1.46 ± 0.12	Vitamin B2	1.92 ± 0.10
Ca	0.84 ± 0.06	Glutamic acid	2.20 ± 0.24	Vitamin B6	1.63 ± 0.23
Mg	1.78 ± 0.21	Histidine	2.89 ± 0.09	Vitamin B12	0.55 ± 0.01
S	4.70 ± 0.13	Isoleucine	2.44 ± 0.13		
Fe	0.07 ± 0.14	Leucine	3.15 ± 0.21		
Si	0.12 ± 0.05	Lysine	2.99 ± 0.02		
Zn	0.21 ± 0.10	Methionine	0.83 ± 0.14		
Mn	0.05 ± 0.09	Proline	1.65 ± 0.10		
Cu	8.90 ± 0.15	Serine	1.74 ± 0.13		
Mo	0.55 ± 0.21	Valine	2.33 ± 0.11		

^¶^ Means of three samples ± SD; *: Awad-Allah et al. [27].

**Table 2:** Bacterial spot disease incidence and severity on sweet pepper leaves after infection, as affected by different treatments of SiO_2_-NPs (ppm), and BCA (%).

SiO_2_-NPs (ppm)	* Mean Bacterial Spot Severity			Mean
	Antagonistic Yeast Extract (%)			
	0	3	5	
**0**	5.06 ^a^	3.96 ^c^	3.58 ^d^	4.23 ^a^
**50**	4.46 ^b^	3.56 ^d^	2.32 ^f^	3.47 ^b^
**100**	2.98 ^e^	2.40 ^f^	1.64 ^i^	2.33 ^c^
**150**	2.06 ^g^	1.80 ^h^	1.20 ^j^	1.70 ^d^
**Mean**	3.68 ^a^	2.95 ^b^	2.18 ^c^	
**LSD_0.05_**	SiO_2_-NPs 0.082	BCA 0.071	Interaction 0.143	

* Disease severity was recorded as the following scale: 1 = symptomless, 2 = a few necrotic spots on a few leaflets, 3 = a few necrotic spots on many leaflets, 4 = many spots with coalescence on few leaflets, 5 = many spots with coalescence on many leaflets, 6 = severe disease and leaf defoliation, and 7 = plant dead. Means followed by the same alphabetical letter(s) in common are not significantly different at *(p* ≤ 0.05).

**Table 3:** Leaf chemical composition (%) of sweet pepper plants as affected by different treatments of SiO_2_-NPs (ppm) and BCA (%) without and with bacterial leaf spot (BLS) infection.

Treatments			Leaf Chemical Composition (%) of Sweet Pepper Plants					
(BLS) Infection	BCA (%)	SiO_2_-NPs (ppm)	N	P	K	Ca	Mg	Si
Without	0	0	3.00 ^o^	0.30 ^o^	2.45 ^n^	1.01 ^o^	0.28 ^n^	0.12 ^op^
		50	3.40 ^mn^	0.37 ^kl^	3.00 ^k^	1.20 ^jk^	0.30 ^lm^	0.33 ^l^
		100	4.10 _h_	0.39 ^ij^	3.32 ^gh^	1.30 ^gh^	0.37 ^gh^	0.42 ^h^
		150	4.60 ^d^	0.46 ^e^	3.71 ^e^	1.40 ^de^	0.44 ^d^	0.46 ^fg^
	3	0	3.55 ^l^	0.35 ^m^	2.90 ^kl^	1.15 ^l^	0.33 ^j^	0.11 ^pg^
		50	3.95 ^i^	0.40 ^hi^	3.25 ^hij^	1.28 ^hi^	0.34 ^j^	0.40 ^ij^
		100	4.30 ^f^	0.42 ^h^	3.60 ^f^	1.38 ^e^	0.42 ^e^	0.47 ^ef^
		150	4.95 ^b^	0.52 ^b^	4.30 ^b^	1.44 ^c^	0.50 ^b^	0.53 ^c^
	5	0	3.85 ^j^	0.41 ^h^	3.20 ^ij^	1.30 ^gh^	0.38 ^fg^	0.12 ^op^
		50	4.20 ^g^	0.43 ^g^	3.55 ^f^	1.34 ^f^	0.39 ^f^	0.45 ^g^
		100	4.70 ^c^	0.50 ^c^	4.00 ^d^	1.47 ^b^	0.48 ^c^	0.51 ^d^
		150	5.09 ^a^	0.55 ^a^	4.58 ^a^	1.54 ^a^	0.55 ^a^	0.60 ^a^
With	0	0	2.70 ^q^	0.21 ^q^	1.81 ^p^	0.85 ^q^	0.22 ^p^	0.10 ^q^
		50	3.00 ^o^	0.30 ^o^	2.40 ^n^	1.00 ^o^	0.24 ^o^	0.26 ^n^
		100	3.34 ^n^	0.35 ^m^	2.80 ^l^	1.13 ^lm^	0.31 ^kl^	0.34 ^l^
		150	3.90 ^ij^	0.40 ^hi^	3.30 ^gh^i	1.26 ^i^	0.37 ^gh^	0.39 ^j^
	3	0	2.90 ^p^	0.25 ^p^	2.28 ^o^	0.96 ^p^	0.28 ^n^	0.13 ^o^
		50	3.45 ^m^	0.32 ^n^	2.85 ^l^	1.08 ^n^	0.29 ^mn^	0.30 ^m^
		100	3.56 ^l^	0.38 ^jk^	3.20 ^ij^	1.22 ^j^	0.36 ^hi^	0.41 ^hi^
		150	4.21 ^g^	0.44 ^f^	3.95 ^d^	1.32 ^fg^	0.45 ^d^	0.48 ^e^
	5	0	3.32 ^n^	0.36 ^lm^	2.65 ^m^	1.11 ^m^	0.32 ^jk^	0.10 ^q^
		50	3.75 ^k^	0.39 ^ij^	3.14 ^j^	1.18 ^k^	0.35 ^i^	0.37 ^k^
		100	4.10 ^h^	0.45 ^ef^	3.38 ^g^	1.29 ^h^	0.39 ^f^	0.46 ^fg^
		150	4.40 ^e^	0.48 ^d^	4.17 ^c^	1.41 ^d^	0.49 ^bc^	0.55 ^b^

Means in each column, followed by the same alphabetical letter(s) in common, are not significantly different at *p* ≤ 0.05.

## Data Availability

The data presented in this study are available on request from the corresponding author.
